# Hsa_circ_0002162 has a critical role in malignant progression of tongue squamous cell carcinoma through targeting miR-33a-5p

**DOI:** 10.1590/1414-431X202010093

**Published:** 2021-03-15

**Authors:** Chunguang Zhang, Yuan Yao, Lei Bi

**Affiliations:** 1 Stomatology Department, North China University of Science and Technology Affiliated Hospital, Tangshan, China

**Keywords:** Hsa_circ_0002162, Tongue squamous cell carcinoma, miR-33a-5p, Cell proliferation, Cell apoptosis, Cell invasion

## Abstract

The aim of this study was to explore the effect of hsa_circ_0002162 on regulating cell proliferation, apoptosis, and invasion, and investigate its potential target microRNA (miRNA) in tongue squamous cell carcinoma (TSCC). Hsa_circ_0002162 expression was detected in human TSCC cell lines and human oral keratinocytes (HOK) cell line. Cell proliferation, apoptosis, invasion, and candidate target miRNA expressions were detected in hsa_circ_0002162 knockdown-treated CAL-27 cells and hsa_circ_0002162 overexpression-treated SCC-9 cells. In the rescue experiment, miR-33a-5p knockdown plasmid was transfected into hsa_circ_0002162 knockdown-treated CAL-27 cells, while miR-33a-5p overexpression plasmid was transfected into hsa_circ_0002162 overexpression-treated SCC-9 cells. Subsequently, cell proliferation, apoptosis, and invasion were detected, and then luciferase reporter assay was performed. Hsa_circ_0002162 expression was increased in human TSCC cell lines SCC-9, CAL-27, HSC-4, and SCC-25 compared with HOK. In CAL-27 cells, hsa_circ_0002162 knockdown inhibited cell proliferation and invasion and promoted apoptosis. In SCC-9 cells, hsa_circ_0002162 overexpression enhanced cell proliferation and invasion and suppressed apoptosis. Furthermore, a negative regulation of hsa_circ_0002162 on miR-33a-5p (but not miR-302b-5p and miR-545-5p) was observed. In the rescue experiment, miR-33a-5p knockdown increased cell proliferation and invasion, and decreased apoptosis in hsa_circ_0002162 knockdown-treated CAL-27 cells, whereas miR-33a-5p overexpression decreased cell proliferation and invasion, but increased apoptosis in hsa_circ_0002162 overexpression-treated SCC-9 cells. The luciferase reporter assay showed the direct binding of hsa_circ_0002162 to miR-33a-5p. In conclusion, hsa_circ_0002162 had an important role in malignant progression of TSCC through targeting miR-33a-5p.

## Introduction

Tongue squamous cell carcinoma (TSCC) is the most common malignancy of the oral cavity, and it is also one of the most lethal head and neck cancers worldwide (1,2). Due to its characteristic silence (progressing from a premalignant state into invasive carcinoma without any specific alarming symptoms), most TSCC patients are in an advanced stage when diagnosed (3). Even with improved treatments (including surgery resection, chemotherapy, and radiotherapy), the prognosis of TSCC patients is still unsatisfactory with the 5-year relative survival rate of 63% (4). Hence, investigation of molecular mechanisms underlying TSCC is necessary to aid in the development of novel therapy targets and improve the prognosis of TSCC patients.

Circular RNAs (circRNAs) are a novel type of endogenous RNAs featuring covalently closed continuous loops, which not only sponge microRNAs (miRNAs) but also interact with RNA-binding proteins (5). Previous studies reveal that multiple circRNAs are differentially expressed in several cancers, and their dysregulation contributes to tumor progression by promoting cell viability, cell mobility, epithelial mesenchymal transformation (EMT), and even cell stemness in various carcinomas (such as TSCC, hepatocellular cancer, and gastric cancer) (6–8). As one of the newly discovered circRNAs, hsa_circ_0002162 has been reported to be highly expressed in TSCC tumor tissues compared to adjacent tissues according to a recent study with high‐throughput sequencing (in three TSCC tissues and adjacent tissues). Further reverse transcription quantitative polymerase chain reaction (RT-qPCR) also showed higher expression of hsa_circ_0002162 in TSCC tumor tissues than adjacent tissues, consistent with the high‐throughput data (9). No more evidence regarding the role of hsa_circ_0002162 in TSCC was found. In addition, miR-33a-5p, miR-302b-5p, and miR-545-5p are reported to be potential targets of hsa_circ_0002162 in TSCC (9), and these three miRNAs have been observed to exert regulatory effects on cell proliferation and cell invasion in multiple cancers (10–12). Thus, we hypothesized that hsa_circ_0002162 might target miR-33a-5p, miR-302b-5p, and/or miR-545-5p to promote TSCC tumorigenesis. Therefore, in the current study, the aim was to explore the effect of hsa_circ_0002162 on regulating cell proliferation, apoptosis, and invasion, and to investigate its potential target miRNA in TSCC cell lines.

## Material and Methods

### Cell culture

Human TSCC cell lines SCC-9, CAL-27, and SCC-25 were bought from American Type Culture Collection (ATCC, USA), and human TSCC cell line HSC-4 was purchased from Japanese Collection of Research Bioresources Cell Bank (JCRB, Japan). Human oral keratinocytes (HOK) was purchased from ScienCell Research Laboratories, Inc. (USA). The SCC-9 and CAL-27 cells were cultured in 90% Dulbecco's modified Eagle's medium (Sigma, USA) supplemented with 10% fetal bovine serum (FBS) (Sigma). The SCC-25 cells were cultured in 90% Dulbecco's modified Eagle medium/nutrient mixture F-12 supplemented with 10% FBS (Sigma). HSC-4 cells were cultured in 90% Eagle's minimum essential medium (Sigma) supplemented with 10% FBS (Sigma). HOK cells were cultured in oral keratinocyte medium (ScienCell, USA). All cells were cultured in incubators with 5% CO2, 95% air at 37°C. The expression of hsa_circ_0002162 in TSCC cells and HOK cells was detected by RT-qPCR.

### Hsa_circ_0002162 plasmid construction and transfection

pGPH1 vector was applied to construct hsa_circ_0002162 knock-down plasmid and circRNA control knock-down plasmid by GenePharma Co., Ltd (China). pCD5-ciR vector was applied to construct hsa_circ_0002162 overexpression plasmid and circRNA control overexpression plasmid by Geneseed Biotech Co., Ltd. (China). Hsa_circ_0002162 knock-down plasmid and circRNA control knock-down plasmid were transfected into CAL-27 cells using HilyMax (Dojindo, Japan), and the cells were divided into Circ(-) cells and NC(-) cells, accordingly. Hsa_circ_0002162 overexpression plasmid and circRNA control overexpression plasmid were transfected into SCC-9 cells using HilyMax (Dojindo), and the cells were divided into Circ(+) cells and NC(+) cells, accordingly. The expression of hsa_circ_0002162 in the four cell lines was evaluated by RT-qPCR at 24 h post transfection.

### Cell proliferation, apoptosis, and invasion measurements

At 0, 24, 48, and 72 h after transfection, Cell Counting kit-8 (Dojindo) was used to perform CCK-8 assay to detect cell proliferation. At 48 h after transfection, Annexin V-FITC apoptosis detection kit (R & D, USA) was used to perform annexin V/propidium iodide (AV/PI) assay to assess cell apoptosis. Furthermore, 24 h after transfection, Matrigel^®^ basement membrane matrix coated chamber (Corning, USA) was used to perform Transwell assay to determine cell invasion ability.

### Target miRNA measurement

According to a previous study, microRNA-33a-5p (miR-33a-5p), microRNA-302b-5p (miR-302b-5p), and microRNA-545-5p (miR-545-5p) are potential targets of hsa_circ_0002162 in TSCC (9). Furthermore, miR-33a-5p, miR-302b-5p, and miR-545-5p play important roles in regulating malignant cell proliferation and invasion in various cancers (10–12). Therefore, to determine the target of has_circ-0002162, the expressions of miR-33a-5p, miR-320b, and miR-545-5p were detected by RT-qPCR at 24 h after transfection.

### Rescue experiments: miR-33a-5p plasmid construction, transfection, and detection

pGCMV/EGFP/miR/inhibitor vector was used to construct miR-33a-5p knock-down plasmid and microRNA (miRNA) control knock-down plasmid by GnenPharma (China). pGCMV/EGFP/miR/blasticidin vector was used to construct miR-33a-5p overexpression plasmid and miRNA control overexpression plasmid. miR-33a-5p knock-down plasmid and miRNA control knock-down plasmid were transfected into Circ(-) cells with HilyMax (Dojindo), which were termed as Circ(-) & miR(-) and Circ(-) & NC(-) cells, respectively. miR-33a-5p overexpression plasmid and miRNA control overexpression plasmid were transfected into Circ(+) cells with HiyMax (Dojindo), which were termed as Circ(+) & miR(+) and Circ(+) & NC(+) cells, respectively. At 24 h after transfection, the expressions of miR-33a-5p and hsa_circ_0002162 were determined by RT-qPCR. Cell proliferation, apoptosis, and invasion were assessed by the methods described in the “Cell proliferation, apoptosis, and invasion measurements” subsection.

### RT-qPCR assay

Total RNA was extracted by TRIzol™ reagent (Invitrogen, USA), and the concentration and purity of RNA were determined by spectrophotometry. For circRNA (but not for miRNA or mRNA), 1 μg total RNA was used for linear RNAs digestion using RNase R enzyme (Epicentre, USA), and subsequently, reverse transcription was performed using PrimeScript™ RT reagent kit (Takara), followed by qPCR using TB Green^®^ Fast qPCR mix (Takara). For miRNA and mRNA detection, 1 μg total RNA was reversely transcribed to cDNA using PrimeScript™ RT reagent kit (Takara), and qPCR was carried out using TB Green^®^ Fast qPCR mix (Takara). According to a previous study (9), hsa_circ_0002162 is identified in TSCC and detected by RT-qPCR. Therefore, we referred to the prior hsa_circ_0002162 primer in this study. Primers are listed in Table 1. Glyceraldehyde-phosphate dehydrogenase (GAPDH) was used as internal reference of circRNA, and U6 was used as internal reference of miRNA. Relative quantification of gene expression was performed by the 2^-△△Ct^ method.

**Table 1:** Primer sequences used in the study.

Gene	Forward primer (5′-3′)	Reverse primer (5′-3′)
hsa_circ_0002162	GGGGCAATGCACTAGAAAAG	AATCGCTCTTCACCTGTTGAT
miR-33a-5p	ACACTCCAGCTGGGGTGCATTGTAGTTGCA	TGTCGTGGAGTCGGCAATTC
miR-302b-5p	ACACTCCAGCTGGGACTTTAACATGGAAGT	TGTCGTGGAGTCGGCAATTC
miR-545-5p	ACACTCCAGCTGGGTCAGTAAATGTTTATT	TGTCGTGGAGTCGGCAATTC
U6	CGCTTCGGCAGCACATATACTA	ATGGAACGCTTCACGAATTTGC
GAPDH	GAGTCCACTGGCGTCTTCAC	ATCTTGAGGCTGTTGTCATACTTCT

### Luciferase reporter assay

Dual-Luciferase^®^ reporter (DLR™) assay system (Promega, USA) was used for Luciferase reporter assay. pGL4 vector (Promega) was used to construct hsa_circ_0002162 wild type (WT) plasmid and mutant type (MT) plasmid. miR-33a-5p overexpression (OE-miR-33a-5p) plasmid, miRNA control overexpression (OE-NC) plasmid, hsa_circ_0002162 WT plasmid, and hsa_circ_0002162 MT plasmid were co-transfected into 293T cells (ATCC) using HilyMax (Dojindo), and the cells were named as WT & OE-NC cells, WT & OE-miR-33a-5p cells, MT & OE-NC cells, and MT & OE-miR-33a-5p cells, respectively. Cells were lysed and firefly luciferase luminescence was detected according to the manufacturer's instructions at 24 h post transfection.

### Statistical analysis

Data are reported as mean and standard deviation. GraphPad Prism software version 7.0 (GraphPad Software Inc., USA) was applied for data analysis and graph making. Comparison between two groups was determined by the unpaired *t*-test, and comparison among more than two groups was determined by one-way analysis of variance (ANOVA) followed by Dunnett's multiple comparisons test. A P value <0.05 was considered as statistically significant.

## Results

### Comparison of hsa_circ_0002162 expression between human TSCC cell lines and HOK cell line

**Figure 1:**
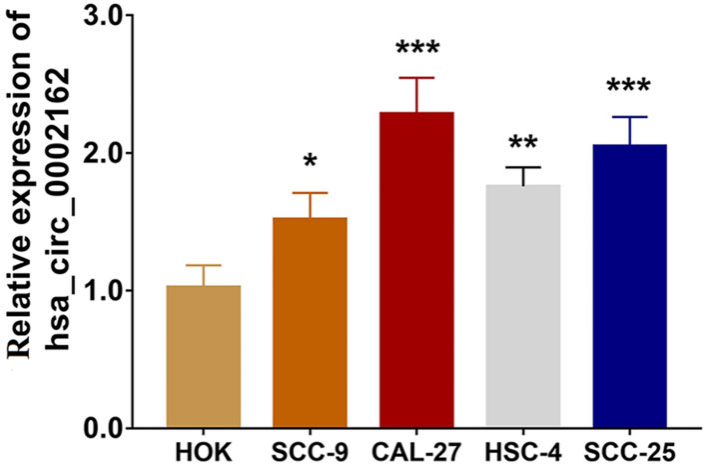
Expression of hsa_circ_0002162 in human tongue squamous cell carcinoma cell lines and human oral keratinocytes (HOK). Data are reported as means±SD. *P<0.05, **P<0.01, ***P<0.001 compared to HOK (ANOVA).

Compared with HOK, hsa_circ_0002162 expression was increased in human TSCC cell lines SCC-9 (P<0.05), CAL-27 (P<0.001), HSC-4 (P<0.01), and SCC-25 (P<0.001) (Figure 1). Considering that hsa_circ_0002162 expression was highest in CAL-27 cells and lowest in SCC-9 cells, these were chosen for the subsequent experiments.

### Hsa_circ_0002162 expression after transfection

**Figure 2:**
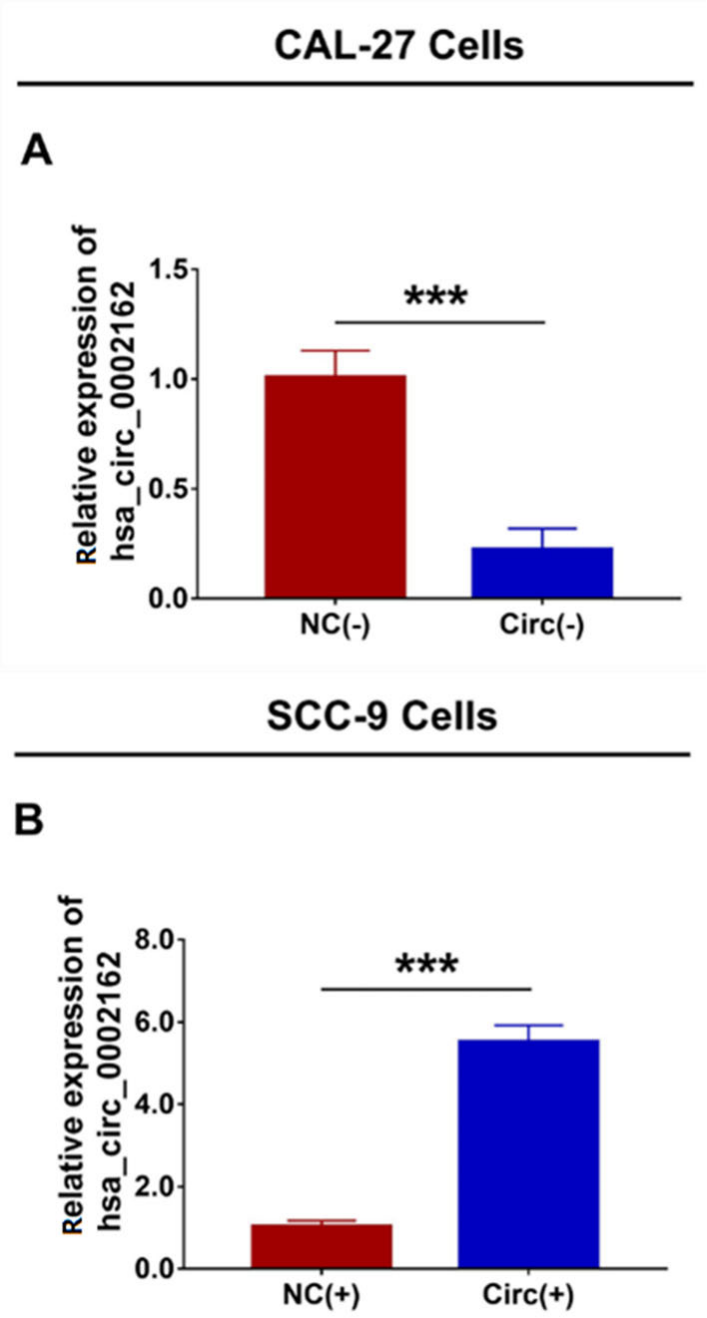
A, Comparison of hsa_circ_0002162 expression between the Circ(-) group and the NC(-) group. **B**, Comparison of hsa_circ_0002162 expression between the Circ(+) group and the NC(+) group. Hsa_circ_0002162 knock-down plasmid and circRNA control knock-down plasmid were transfected into CAL-27 cells and then divided into Circ(-) cells and NC(-) cells, accordingly. Hsa_circ_0002162 overexpression plasmid and circRNA control overexpression plasmid were transfected into SCC-9 cells and then divided into Circ(+) cells and NC(+) cells, accordingly. Data are reported as means±SD. ***P<0.001 (*t*-test). circ: circRNAs; NC: negative control.

In CAL-27 cells, hsa_circ_0002162 expression was decreased in the Circ(-) group compared to the NC(-) group (P<0.001) after transfection (Figure 2A). In SCC-9 cells, hsa_circ_0002162 expression was increased in the Circ(+) and NC(+) groups (P<0.001) after transfection (Figure 2B). In addition, in CAL-27 cells, hsa_circ_0002162 expression was increased in the Circ(+)and NC(+) groups (P<0.001) after transfection (Figure S1A). In SCC-9 cells, hsa_circ_0002162 expression was decreased in the Circ(-) group compared to the NC(-) group (P<0.001) after transfection (Figure S1B). These data suggested successful transfection in CAL-27 cells and SCC-9 cells.

### Function of hsa_circ_0002162 on cell proliferation, apoptosis, and invasion

**Figure 3:**
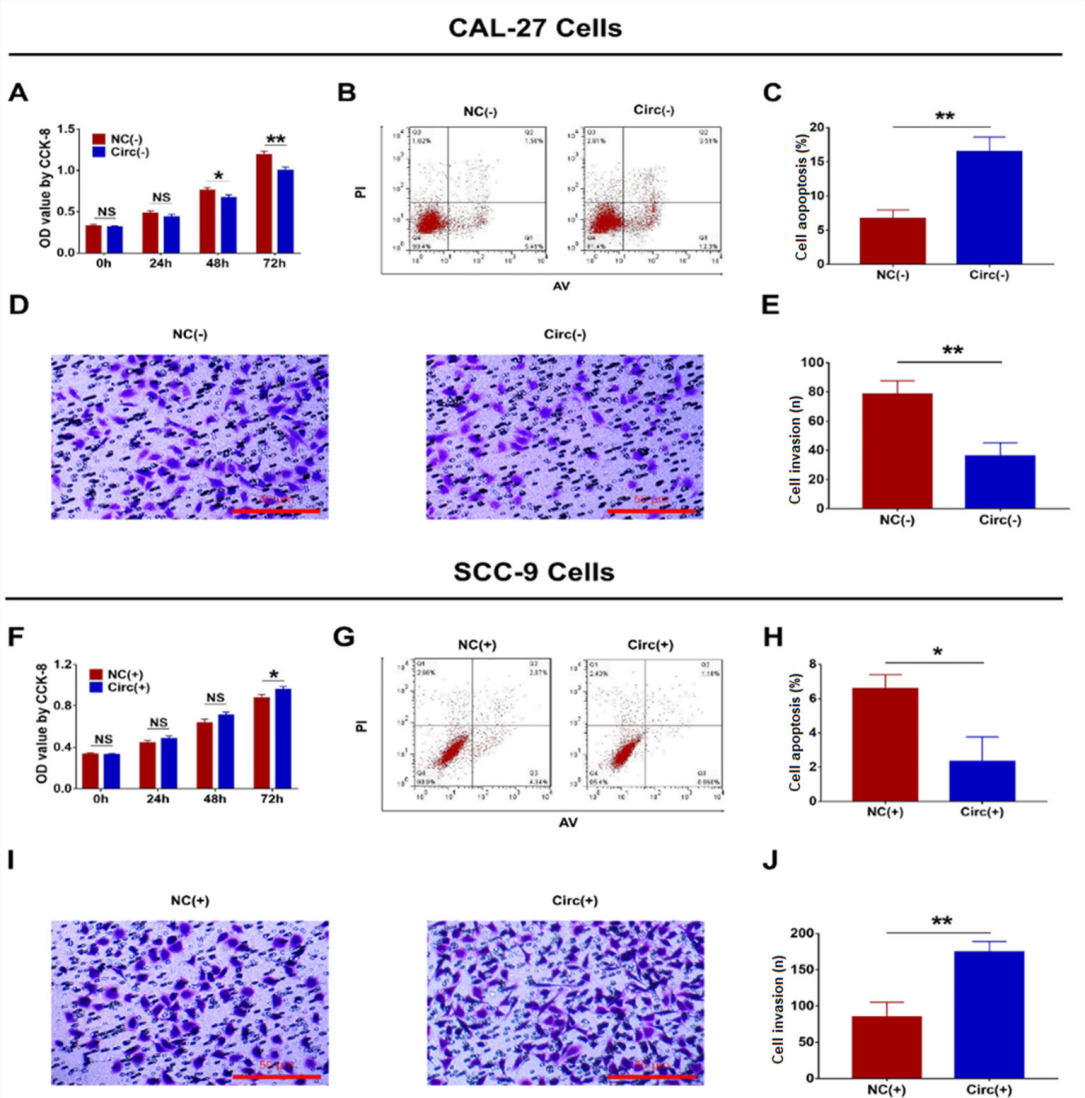
Comparison of cell proliferation (**A**), apoptosis (**B** and **C**), and invasion (**D** and **E**) between the Circ(-) group and the NC(-) group (scale bar: 50 μm). Comparison of cell proliferation (**F**), apoptosis (**G** and **H**), and invasion (**I** and **J**) between the Circ(+) group and the NC(+) group (scale bar: 50 μm). Hsa_circ_0002162 knock-down plasmid and circRNA control knock-down plasmid were transfected into CAL-27 cells and then divided into Circ(-) cells and NC(-) cells, accordingly. Hsa_circ_0002162 overexpression plasmid and circRNA control overexpression plasmid were transfected into SCC-9 cells and then divided into Circ(+) cells and NC(+) cells, accordingly. Data are reported as means±SD. *P<0.05, **P<0.01 (*t*-test). NS: not significant; NC: negative control.

**Figure 4:**
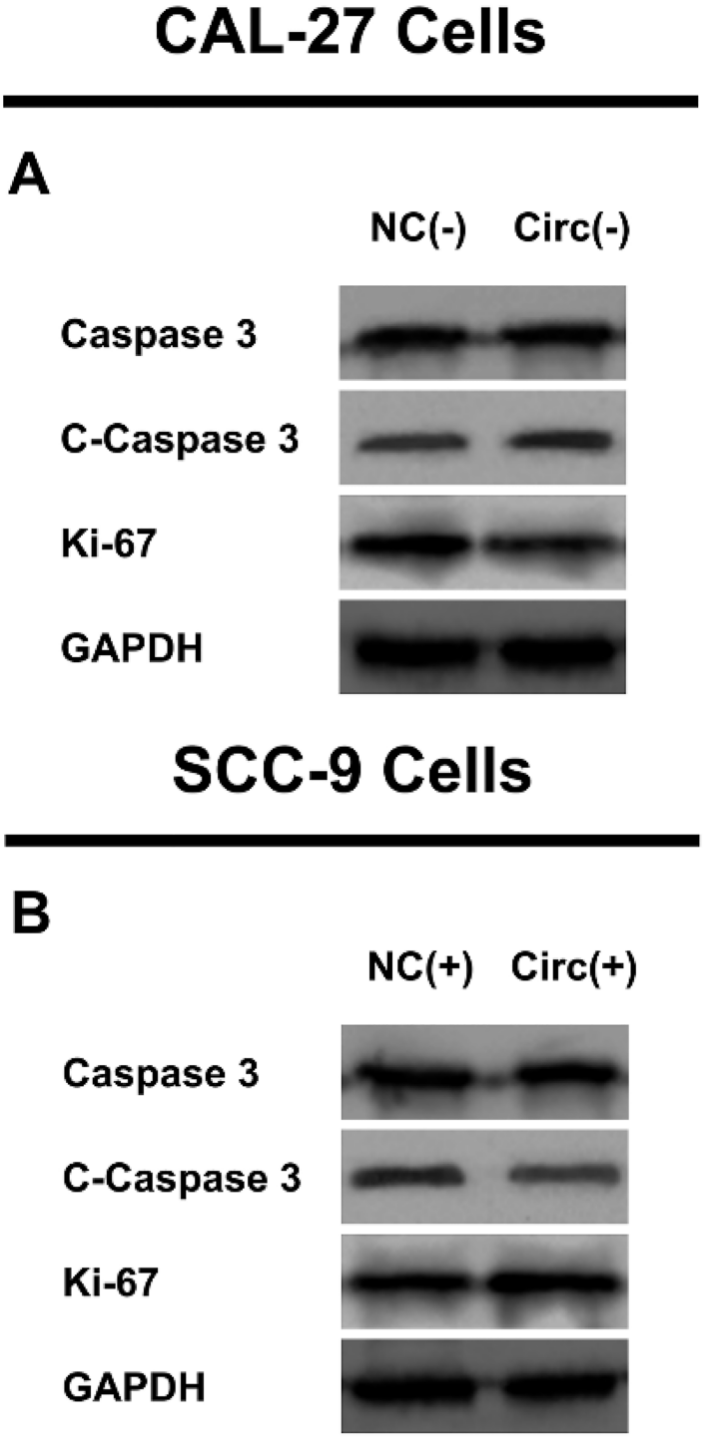
Regulation of hsa_circ_0002162 on proliferation/apoptosis protein expressions in CAL-27 (**A**) and SCC-9 (**B**) cells. Hsa_circ_0002162 knock-down plasmid and circRNA control knock-down plasmid were transfected into CAL-27 cells and then divided into Circ(-) cells and NC(-) cells, accordingly. Hsa_circ_0002162 overexpression plasmid and circRNA control overexpression plasmid were transfected into SCC-9 cells and then divided into Circ(+) cells and NC(+) cells, accordingly. NC: negative control; GAPDH: glyceraldehyde-phosphate dehydrogenase.

In CAL-27 cells, cell proliferation was inhibited in the Circ(-) group compared to the NC(-) group at 48 h (P<0.05) and 72 h (P<0.01) (Figure 3A) and cell apoptosis was promoted in the Circ(-) group compared to the NC(-) group at 48 h (P<0.01) (Figure 3B and C). Meanwhile, cell invasion was suppressed in the Circ(-) group compared to the NC(-) group at 24 h (P<0.01) (Figure 3D and E). In SCC-9 cells, cell proliferation was enhanced in the Circ(+) group compared to the NC(+) group at 72h (P<0.05) (Figure 3F) and cell apoptosis was repressed in the Circ(+) group compared to the NC(+) group at 48 h (P<0.05) (Figure 3G and H). In addition, cell invasion was increased in the Circ(+) group compared to the NC(+) group at 24 h (P<0.01) (Figure 3I and J). In addition, the expression of proliferation protein ki-67 was decreased but the expression of apoptosis protein C-caspase 3 was increased in the Circ(-) group compared to the NC(-) group (Figure 4A). However, the opposite trend was shown in the Circ(+) group compared to the NC(+) group (Figure 4B).

### Function of hsa_circ_0002162 on regulation of miR-33a-5p, miR-302b-5p, and miR-545-5p expressions

**Figure 5:**
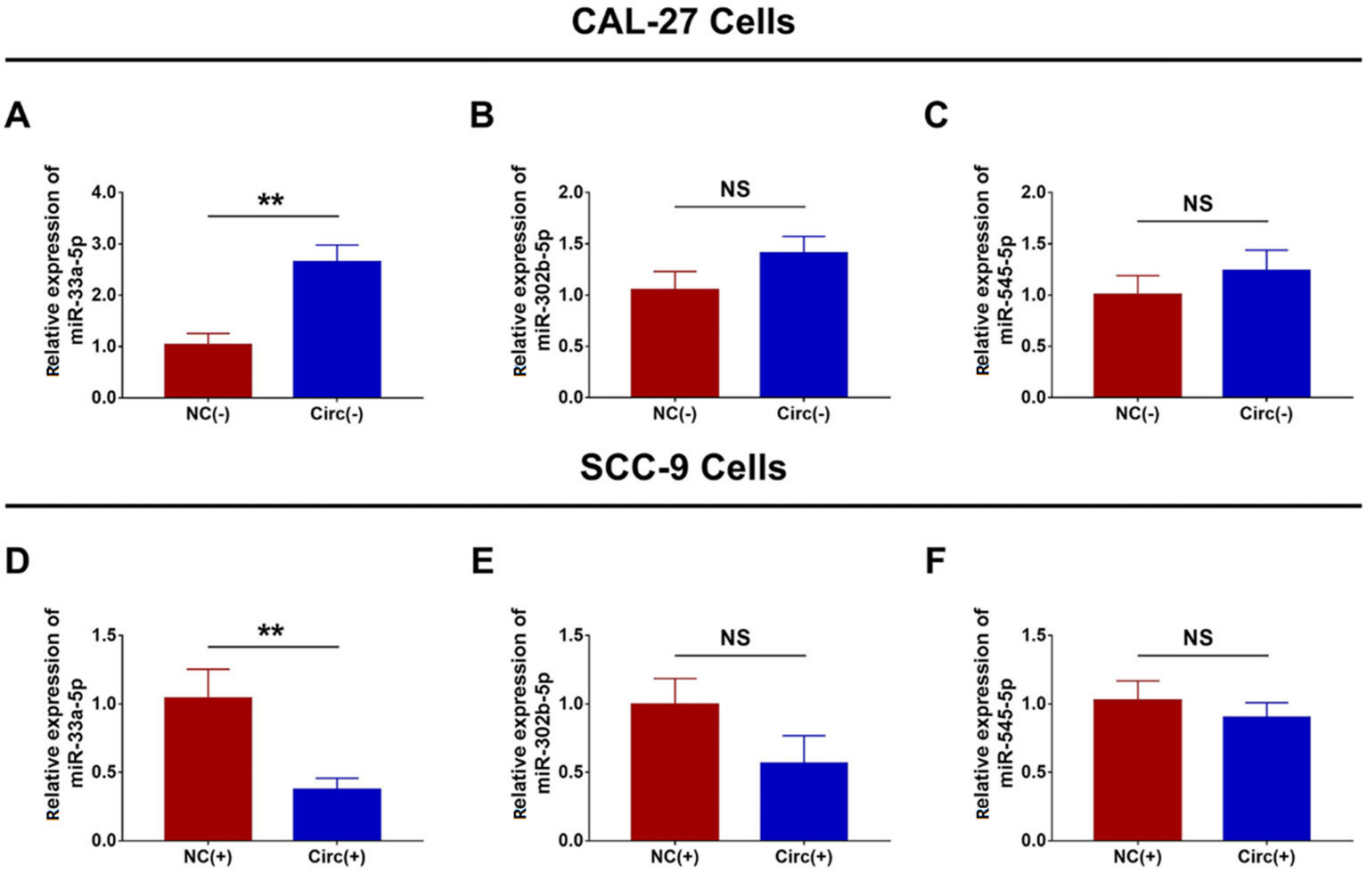
Comparison of **A**, miR-33a-5p, **B**, miR-302b-5p, and **C**, miR-545-5p expressions between the Circ(-) group and the NC(-) group. Comparison of **D**, miR-33a-5p, **E**, miR-302b-5p, and **F**, miR-545-5p expressions between the Circ(+) group and the NC(+) group. Hsa_circ_0002162 knock-down plasmid and circRNA control knock-down plasmid were transfected into CAL-27 cells and then divided into Circ(-) cells and NC(-) cells, accordingly. Hsa_circ_0002162 overexpression plasmid and circRNA control overexpression plasmid were transfected into SCC-9 cells and then divided into Circ(+) cells and NC(+) cells, accordingly. Data are reported as means±SD. **P<0.01 (*t*-test). NS: not significant; miR: microRNA; Circ: circRNA; NC: negative control.

In CAL-27 cells, miR-33a-5p expression was increased in the Circ(-) group compared to the NC(-) group (P<0.01) (Figure 5A), while no difference in miR-302b-5p (Figure 5B) and miR-545-5p (Figure 5C) expressions was found between the two groups (both P>0.05). In SCC-9 cells, miR-33a-5p expression was decreased in the Circ(+) group compared to the NC(+) group (P<0.01) (Figure 5D), while there was no difference in miR-302b-5p (Figure 5E) and miR-545-5p (Figure 5F) expressions between the two groups (both P>0.05). These data suggested the negative regulation of hsa_circ_0002162 on miR-33a-5p in TSCC cells.

### Function of hsa_circ_0002162 on miR-33a-5p expressions in the rescue experiment

**Figure 6:**
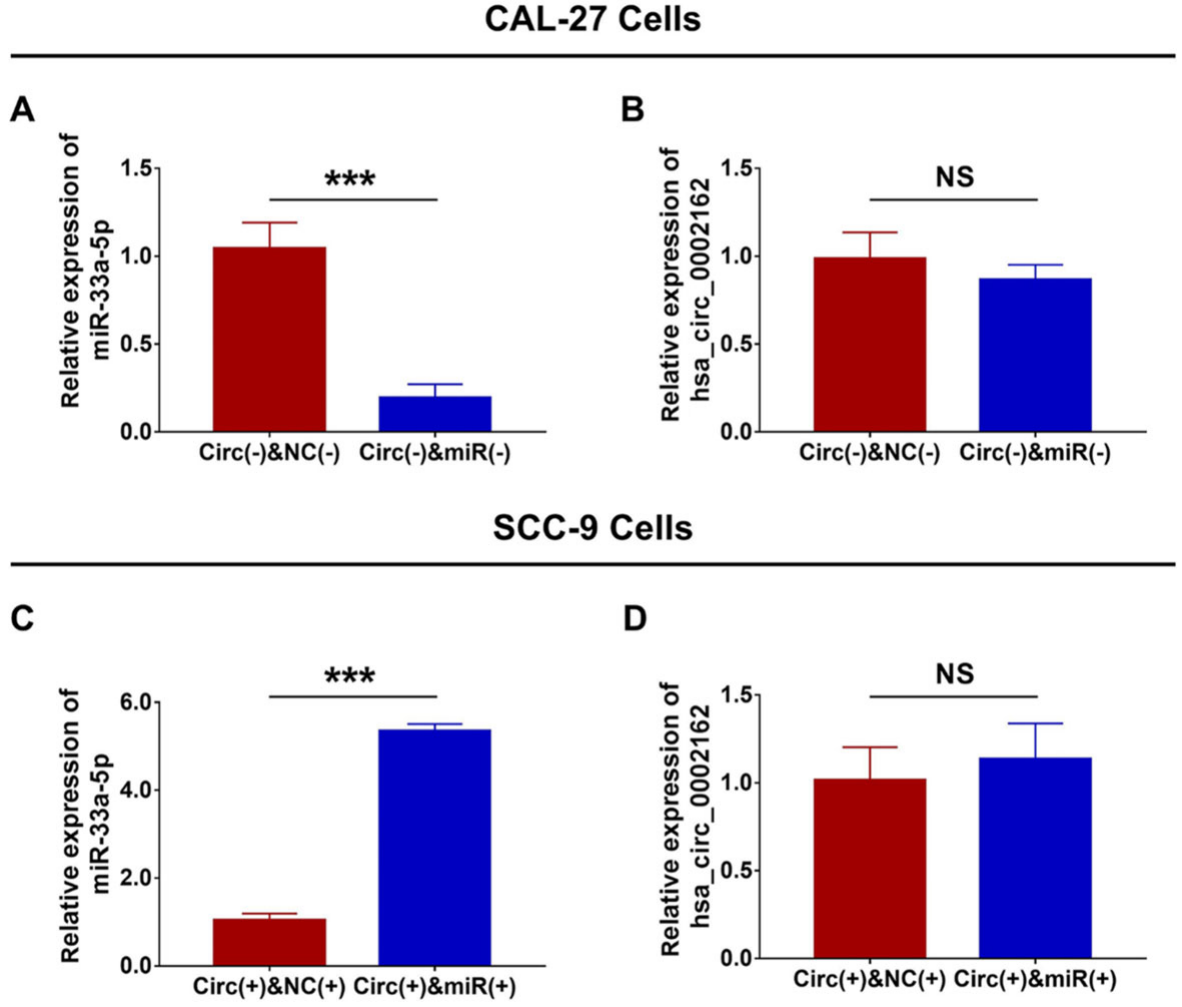
Comparison of **A**, miR-33a-5p and **B**, hsa_circ_0002162 expressions between the Circ(-) & miR(-) group and the Circ(-) & NC(-) group. Comparison of **C**, miR-33a-5p and **D**, hsa_circ_0002162 expressions between the Circ(+) & miR(+) group and the Circ(+) & NC(+) group. Data are reported as means±SD. ***P<0.001 (*t*-test). NS: not significant; miR: microRNA; Circ: circRNA; NC: negative control.

In CAL-27 cells, miR-33a-5p expression was decreased in the Circ(-) & miR(-) group compared to the Circ(-) & NC(-) group (P<0.001) (Figure 6A), while there was no difference in hsa_circ_0002162 expression between the two groups (P>0.05) (Figure 6B). In SCC-9 cells, miR-33a-5p expression was increased in the Circ(+) & miR(+) group compared to the Circ(+) & NC(+) group (P<0.001) (Figure 6C), while no difference was discovered in hsa_circ_0002162 expression between the two groups (P>0.05) (Figure 6D). These data indicated that miR-33a-5p knock-down plasmid and miR-33a-5p overexpression plasmid were successfully transfected into Circ(-) cells and Circ(+) cells, respectively in the rescue experiment, and miR-33a-5p did not affect hsa_circ_0002162 reversely in TSCC cells.

### Function of hsa_circ_0002162 on cell proliferation, apoptosis, and invasion in the rescue experiment

**Figure 7:**
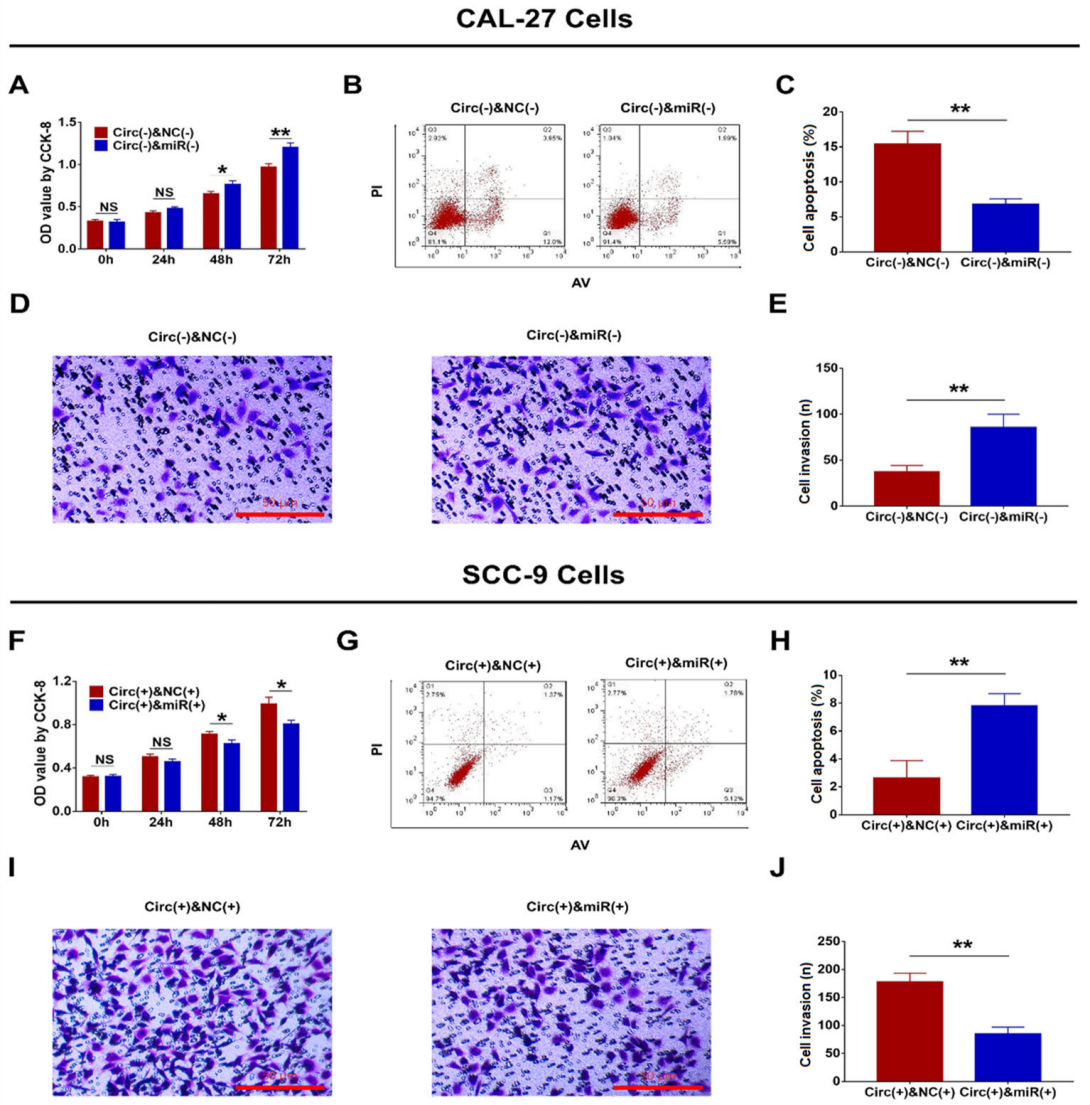
Comparison of cell proliferation (**A**), apoptosis (**B** and **C**), and invasion (**D** and **E**) between the Circ(-) & miR(-) group and the Circ(-) & NC(-) group (scale bar: 50 μm). Comparison of cell proliferation (**F**), apoptosis (**G** and **H**), and invasion (**I** and **J**) between the Circ(+) & miR(+) group and the Circ(+) & NC(+) group (scale bar: 50 μm). Data are reported as means±SD. *P<0.05, **P<0.01 (*t*-test). NS: not significant; NC: negative control.

**Figure 8:**
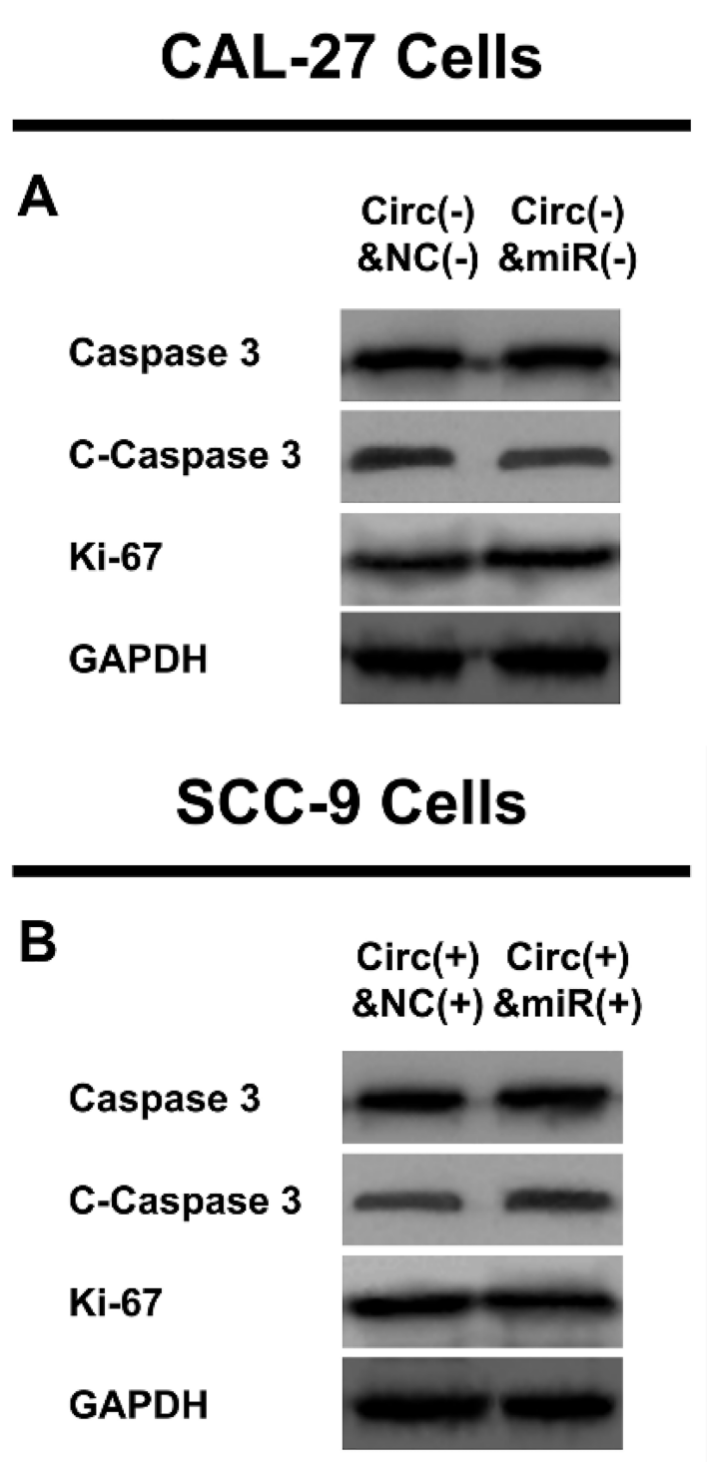
A,Effect of miR-33a-5p on proliferation/apoptosis protein expressions in hsa_circ_0002162 dysregulated CAL-27 cells. **B**, Effect of miR-33a-5p on proliferation/apoptosis protein expressions in hsa_circ_0002162 dysregulated SCC-9 cells. NC: negative control; GAPDH: glyceraldehyde-phosphate dehydrogenase.

In CAL-27 cells, cell proliferation was increased in the Circ(-) & miR(-) group compared to the Circ(-) & NC(-) group at 48 h (P<0.05) and 72 h (P<0.01) (Figure 7A), and cell apoptosis was inhibited in the Circ(-) & miR(-) group compared to the Circ(-) & NC(-) group at 48 h (P<0.01) (Figure 7B and C). Furthermore, cell invasion was promoted in the Circ(-) & miR(-) group compared to the Circ(-) & NC(-) group at 24 h (P<0.01) (Figure 7D and E). In SCC-9 cells, cell proliferation was repressed in the Circ(+) & miR(+) group compared to the Circ(+) & NC(+) group at 48 h (P<0.05) and at 72 h (P<0.05) (Figure 7F), whereas cell apoptosis was enhanced in the Circ(+) & miR(+) group compared to the Circ(+) & NC(+) group at 48 h (P<0.01) (Figure 7G and H). Cell invasion was reduced in the Circ(+) & miR(+) group compared to the Circ(+) & NC(+) group at 24 h (P<0.01) (Figure 7I and J). In addition, the expression of proliferation protein ki-67 was increased but the expression of apoptosis protein C-caspase 3 was decreased in in the Circ(-) & miR(-) group compared to the Circ(-) & NC(-) group (Figure 8A). However, the opposite trend was shown in the Circ(+) & miR(+) group compared to the Circ(+) & NC(+) group (Figure 8B).

### Luciferase reporter assay

**Figure 9:**
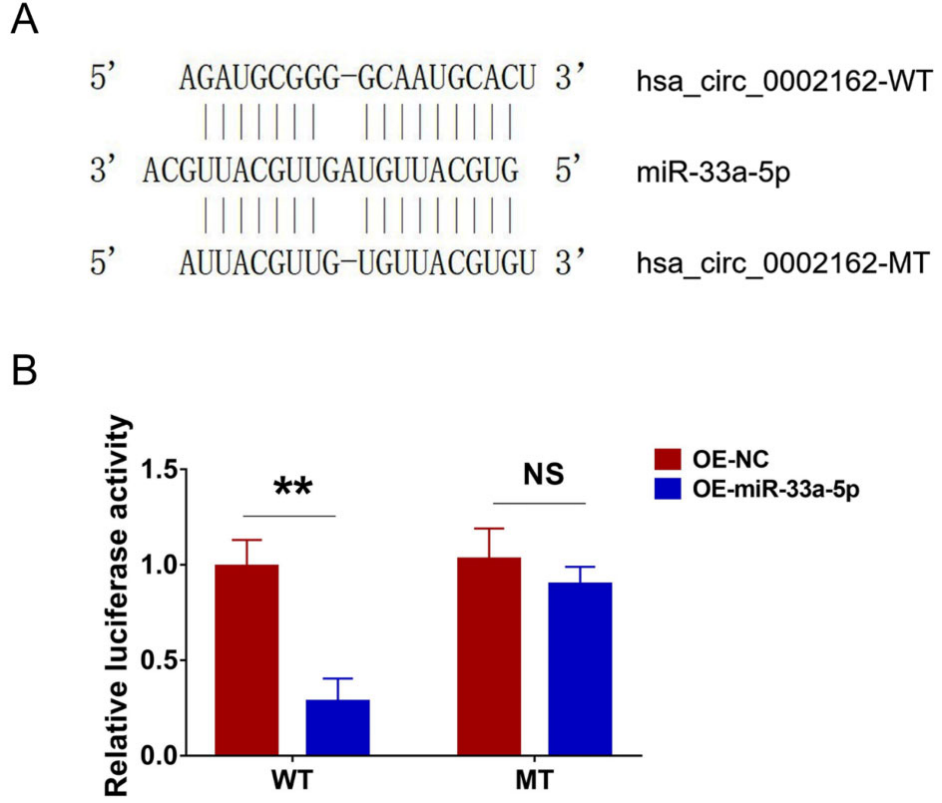
**A**, Designed sequences between hsa_circ_0002162 WT/MT and miR-33a-5p. **B**, Comparison of the relative luciferase activity between WT & OE-miR-33a-5p group and WT & OE-NC group, as well as between MT & OE-miR-33a-5p group and MT & OE-NC group. Data are reported as means±SD. **P<0.01 (*t*-test). NS: not significant; NC: negative control; WT: wild type; MT: mutant type; miR: microRNA; circ: circRNA; OE: overexpression.

Hsa_circ_0002162 had a strong potential to bind miR-33a-5p, and the designed sequences between hsa_circ_0002162 WT/MT and miR-33a-5p are shown (Figure 9A). Relative luciferase activity was reduced in the WT & OE-miR-33a-5p group compared to the WT & OE-NC group (P<0.01), but there was no difference in the relative luciferase activity between the MT & OE-miR-33a-5p group and the MT & OE-NC group (P>0.05) (Figure 9B).

## Discussion

In the present study, hsa_circ_0002162 was highly expressed in human TSCC cell lines SCC-9, CAL-27, HSC-4, and SCC-25 compared with HOK, and it promoted cell proliferation and invasion and repressed cell apoptosis in TSCC cell lines. Interestingly, hsa_circ_0002162 contributed to TSCC tumorigenesis through targeting miR-33a-5p.

CircRNAs are a type of RNA featured by covalent closed loops formed by back-splicing, which play key regulatory roles in pathological processes of various carcinomas, including digestive cancers (7,13,14). For instance, an interesting study reveals that silencing of circ-DONSON (circbase ID: hsa_circ_0004339) represses cell proliferation, migration, and invasion, and enhances apoptosis via recruiting the nucleosome remodeling factor (NURF) complex to initiate SOX4 expression in gastric cancer (7). In addition, silencing of hsa_circ_0067934 represses cell proliferation, inhibits migration, and blocks cell cycle in esophageal squamous cell carcinoma (ESCC) (13). Furthermore, circ_001569 facilitates cell growth, migration, and invasion through sponging miR-411-5p and miR-432-5p in hepatocellular carcinoma (14).

In TSCC, limited information was found in only two previous studies. One interesting study shows that knockdown of circ_0001742 serves as a competing endogenous RNA (ceRNA) to mediate the miR-431-5p/activating transcription factor 3 (ATF3) axis, thereby suppressing cell proliferation, cell migration, cell invasion, and EMT in TSCC cells (15). The other study showed that hsa_circ_0001742 inhibition suppresses cell proliferation, invasion, and EMT processes through targeting the miR-634/ras-related protein Rab-1A (RAB1A) pathway in TSCC (8). Taken together, multiple circRNAs play important roles in the pathology of various carcinomas, including TSCC.

Hsa_circ_0002162 is one of the newly discovered circRNAs. One recent study performed the high‐throughput sequencing to screen circRNA expression profiles from three TSCC tissues and adjacent tissues, and then carried out RT-qPCR for validation of several circRNAs expression profiles, which revealed that hsa_circ_0002162 is highly expressed in TSCC tumor tissues compared to adjacent tissues (9). Based on above information, we hypothesized that has-circ_0002162 might contribute to tumor progression in TSCC. The possible explanations for our results were as follows: i) hsa_circ_0002162 might mediate multiple genes or pathways (such as miR-33a-5p, miR-634/RAB1A pathway (seen as the role of hsa_circ_0001742 in TSCC) [8], or miR-431-5p/ATF3 axis (seen as the role of circ_0001742 in TSCC) [15]) to promote tumorigenesis in TSCC; hence, TSCC cell lines were characterized by increased expression of hsa_circ_0002162. ii) hsa_circ_0002162 might negatively regulate several miRNAs (including miR-33a-5p, miR-411-5p, and miR-432-5p (seen as the role of circ_001569 in hepatocellular carcinoma) [14]) to accelerate cell proliferation and invasion while repressing cell apoptosis in TSCC.

miRNAs (with a length of 21-23 nucleotides) are highly conserved non-coding small RNA molecules with the biological ability to bind to complementary sequences in the 3-untranslated regions of their target mRNAs and induce mRNA degradation or translational repression (16,17). Previous studies have identified several miRNAs with important roles in tumor progression of various carcinomas (18–20). For instance, miR-33a-5p inhibits paraneoplastic Ma family (PNMA) to promote cell proliferation and EMT by activating the Wnt/β-catenin pathway in hepatocellular carcinoma (18). miR-302b suppresses tumor growth and transcription factors protein expression through targeting erb-b2 receptor tyrosine kinase 4 (ERBB4), interferon regulatory factor 2 (IRF2), and Cxc chemokin receptor 4 (CXCR4) in esophageal cancer (19). miR-545 suppresses cell proliferation but accelerates cell apoptosis via targeting retinoic acid-inducible gene-I (RIG-I) in pancreatic ductal adenocarcinoma (20). Considering that miR-33a-5p, miR-302b-5p, and miR-545-5p are reported to be potential targets of hsa_circ_0002162 in TSCC (9), we hypnotized that hsa_circ_0002162 might target miR-33a-5p, miR-302b-5p, and/or miR-545-5p to promote TSCC tumorigenesis. We found that hsa_circ_0002162 negatively regulated miR-33a-5p, but did not affect miR-320b and miR-545-5p, and the rescue experiment showed that hsa_circ_0002162 accelerated cell proliferation and invasion, and suppressed cell apoptosis via targeting miR-33a-5p in TSCC cells. These data provided a new perspective for understanding the underlying mechanism of hsa_circ_0002162 in TSCC.

In conclusion, hsa_circ_0002162 induced apoptosis and decreased cell viability by suppressing miR-33a-5p. The findings may provide a novel perspective on circRNA and lay a foundation for future research of potential roles of circRNA in TSCC.

## Supplementary Material

Click here to view [pdf].
